# Effects of Fe and Mn Deficiencies on the Root Protein Profiles of Tomato (*Solanum lycopersicum*) Using Two-Dimensional Electrophoresis and Label-Free Shotgun Analyses

**DOI:** 10.3390/ijms23073719

**Published:** 2022-03-28

**Authors:** Laura Ceballos-Laita, Daisuke Takahashi, Matsuo Uemura, Javier Abadía, Ana Flor López-Millán, Jorge Rodríguez-Celma

**Affiliations:** 1 Plant Stress Physiology Group, Plant Nutrition Department, Aula Dei Experimental Station, CSIC, 50059 Zaragoza, Spain; ceballos.laita@gmail.com (L.C.-L.); jabadia@eead.csic.es (J.A.); anaflorlopez@gmail.com (A.F.L.-M.); 2 United Graduate School of Agricultural Sciences, Iwate University, Morioka 020-8550, Japan; dtakahashi@mail.saitama-u.ac.jp (D.T.); uemura@iwate-u.ac.jp (M.U.); 3 Department of Plant-Bioscience, Faculty of Agriculture, Iwate University, Morioka 020-8550, Japan

**Keywords:** root, shotgun proteomics, Mn deficiency, Fe deficiency, tomato, 2-DE

## Abstract

Iron (Fe) and manganese (Mn) are two essential elements for plants that compete for the same uptake transporters and show conflicting interactions at the regulatory level. In order to understand the differential response to both metal deficiencies in plants, two proteomic techniques (two-dimensional gel electrophoresis and label-free shotgun) were used to study the proteome profiles of roots from tomato plants grown under Fe or Mn deficiency. A total of 119 proteins changing in relative abundance were confidently quantified and identified, including 35 and 91 in the cases of Fe deficiency and Mn deficiency, respectively, with 7 of them changing in both deficiencies. The identified proteins were categorized according to function, and GO-enrichment analysis was performed. Data showed that both deficiencies provoked a common and intense cell wall remodelling. However, the response observed for Fe and Mn deficiencies differed greatly in relation to oxidative stress, coumarin production, protein, nitrogen, and energy metabolism.

## 1. Introduction

Iron (Fe) and manganese (Mn) are essential metals for all living organisms [1]. In plants, both metals have important roles in fundamental processes such as respiration and photosynthesis, among others [2,3]. In addition, Fe is involved in structural processes, participates in Fe–sulfur clusters, and acts as a cofactor in heme and other Fe-binding sites of proteins [4,5]. On the other hand, Mn participates in the water-splitting site of photosystem II and acts as a cofactor in numerous metabolic processes, forming part of enzymes such as oxalate oxidase, Mn-SOD, and RNA polymerase [6,7].

Despite the fact that most agricultural soils are rich in these metal nutrients, their bioavailability depends on the characteristics of the soil and is often limited, with pH being the most important parameter. In soils with alkaline pH, Fe and Mn occur in oxidized chemical species that are not directly available to plants because of their low solubility [1,8]. Approximately 30% of the arable lands in the world present deficiencies in these metals, with a particular incidence in some areas of Australia, the United States, Southern Europe, North Africa, and Asia [9,10,11]. Iron and Mn deficiencies are particularly relevant in the Mediterranean area, where they affect both the plant growth and the quality of the fruits in many crops [12,13,14,15].

When grown under Fe and Mn deficiencies, plants activate various adaptation responses to increase metal uptake and transport and remobilize stored metals. For Fe uptake by root cells, dicots and non-graminaceous monocot plants such as tomato (*Solanum lycopersicum*) use a set of mechanisms called Strategy I, or reducing strategy, as opposed to the chelation strategy, or Strategy II, used by graminaceous plant species. [16,17]. In the Strategy I species *S. lycopersicum*, these mechanisms are controlled by the transcription factor FER (ortholog to FIT, FER-like Iron deficiency induced Transcription factor, in *Arabidopsis*), which is overexpressed and increases Fe uptake efficiency upon Fe-deficient conditions [18,19,20,21]. Strategy I-associated processes include increases in the activity of the ferric chelate reductase in the root epidermis, which reduces Fe(III) to Fe(II) [22], increases in the expression of *IRT1* (*Iron-Regulated Transporter 1*), which transports Fe(II) into the root cells [4,23], and often increases in the H^+^-ATPase activity, which extrudes protons from roots into the rhizosphere and decreases the pH of the soil, thus increasing Fe solubility. In addition, some Strategy I species release substances synthesized in the roots, such as phenolic compounds (coumarin-type) and flavins, that are able to reduce or chelate Fe under conditions of Fe deficiency [24,25,26]. Much less is known about the Mn deficiency response in plants, which often occurs as a latent disorder. An increase in the frequency of root hairs can be observed as a response to Mn deficiency [27], and in severe cases, root tips develop necrosis [28]. Mn acquisition is mediated mainly by NRAMP (Natural Resistance-Associated Macrophage Protein) transporters that are highly conserved across various organisms [29]. A number of different transporters have been described to mediate Mn trafficking, including the involvement of intracellular vesicles [30,31]. However, little information is known about how Mn homeostasis is regulated, although some data support a strong post-transcriptional regulation [32]. Recently, key post-translational regulators have been found to control NRAMP1 intracellular localization [33,34] and, therefore, to modulate Mn uptake, but no transcriptional regulators or sensing proteins have been found to date.

There is a certain level of antagonism between Fe and Mn homeostasis in plants [35], with demonstrated interference in the uptake of one metal by the other [36]. Metal transporters often have broad specificity, working with many divalent cations, and therefore Fe and Mn share several of them. This lack of specificity implies that a disorder in one of these essential metal micronutrients could affect metal homeostasis by changing the availability of the other. The IRT1 transporter, the main Fe entry to the cell, belongs to a ZRT/IRT-like protein transporter family, which mediates transplasma membrane transport of several transition metals, including Mn. Indeed, mutations in this transporter can be designed to affect its metal selectivity, favouring its specificity for Fe, Zn, or Mn [37]. It has been suggested that Mn deficiency could also increase the expression of HvIRT1 transporter in barley [38]. Recently, it has been demonstrated that the presence of metals others than Fe (e.g., Mn) influence IRT1 subcellular localization, further confirming the interplay between Fe and Mn homeostasis. Furthermore, studies in *Arabidopsis* have shown that members of the NRAMP transporter family are critical in remobilizing vacuolar Mn and Fe in leaves, highlighting again the interaction between both nutrients [39].

A recent study on the protein profiles of xylem sap from *S. lycopersicum*, using label-free shotgun analysis, showed that Fe and Mn deficiencies caused changes in the relative abundance of a similar percentage of proteins, but the trend of these changes was opposite, suggesting differences in the regulation of homeostasis despite sharing some of the metal uptake mechanisms [40]. Given the relevance of both Fe and Mn deficiencies in agriculture, the aim of this work was to elucidate the effects of both deficiencies on the root protein profile in *S. lycopersicum*, using two-dimensional electrophoresis (2-DE) and shotgun analysis to increase coverage, to complete the knowledge available on the mechanisms occurring in response to these nutritional stresses. In order to highlight the similarities and differences between the Fe and Mn deficiency responses, both deficiencies were studied separately, and then the results were compared.

## 2. Results

### 2.1. Effects of Fe and Mn Deficiencies on Leaf Pigments and Metal Concentrations

Tomato plants grown without Fe showed deficiency symptoms as soon as five days after the treatment onset. Visual symptoms at sampling time (eight days) included marked chlorosis in young, expanded leaves. Accordingly, the SPAD values in these leaves were significantly lower than those measured in control plants (Table 1). Total chlorophyll (Chl) in young leaves decreased approximately by 73% in Fe-deficient plants, whereas the Chl a/Chl b ratio did not change (Table 1). The iron shortage resulted in other changes in the leaf pigment composition. The concentrations of antheraxanthin (A) and zeaxanthin (Z) were higher (2.2- and 14.2-fold, respectively) in Fe-deficient plants when compared with those in the controls, whereas those of neoxanthin, violaxanthin (V), and lutein decreased by 80, 74, and 66%, respectively. The concentration of taraxanthin was very low and did not change with the Fe status. The total concentration of violaxanthin cycle pigments (V + A + Z) was 49% lower in plants grown with Fe deficiency when compared with the controls. Consequently, the (A + Z)/(V + A + Z) ratio increased markedly with Fe deficiency, from 0.06 to 0.53.

To achieve Mn-deficiency, plants were grown with zero Mn from germination. At sampling time, young leaves in Mn-deficient plants showed interveinal chlorosis with a “checkered” pattern, resulting in SPAD values 30% lower than those in the control plants (Table 1). Pigment concentrations in Mn-deficient plants presented more moderate changes when compared with Fe-deficient ones. Total Chl decreased approximately by 32%, whereas the Chl a/ Chl b ratio remained constant (Table 1). The concentrations of antheraxanthin (A) and zeaxanthin (Z) were slightly higher (1.5 and 1.3-fold, respectively) in leaves of Mn-deficient plants when compared with those in the controls, whereas those of neoxanthin, violaxanthin (V), and lutein decreased between 43 and 45%. On the other hand, the concentration of taraxanthin was significantly higher (9.6-fold) in Mn-deficient plants when compared with the controls. The total amount of violaxanthin cycle pigments (V + A + Z) was lower (38%) in leaves from plants grown in Mn deficiency than in the controls. In consequence, the (A + Z)/ (V + A+ Z) ratio increased only moderately with Mn deficiency, from 0.06 to 0.14, when compared with control plants.

**Figure 1:**
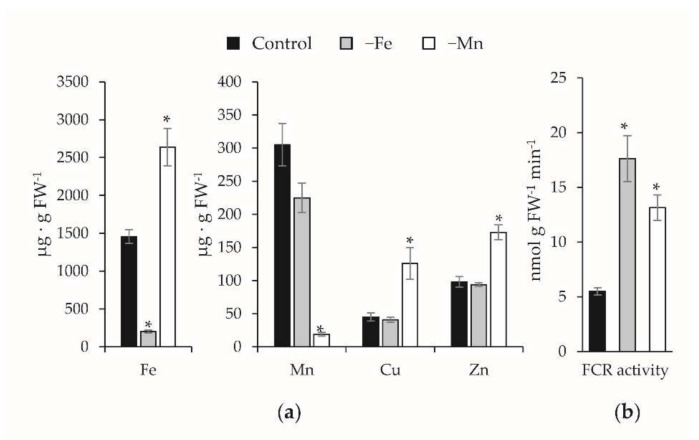
Effect of Fe and Mn deficiency on the root metal concentration (**a**) and Fe reductase (FCR) activity (**b**) (*, *t*-test *p* ≤ 0.05).

The Fe concentration in roots of Fe-deficient plants was 86% lower than that found in the controls, whereas no significant differences were observed in the concentrations of Mn, Cu, and Zn (Figure 1a). On the other hand, the concentration of Mn was 94% lower in roots of Mn-deficient plants than in control ones. Interestingly, the concentration of Fe in the root of Mn-deficient plants was nearly twice that in control plants, and significant increases were also observed in the root concentrations of Cu and Zn. This increase in other metals under Mn deficiency can be due to nonspecific transport by induced divalent metal transporters. The fact that such metal accumulations could lead to cellular oxidative stress and be responsible in part of the Mn deficiency symptoms deserves further studies. No differences were found in the macronutrient concentrations (Ca, Mg, and K; Table S1).

The Fe reductase activity in roots from plants grown in Fe-deficient conditions was 3.2-fold higher than that measured in roots from control plants (Figure 1b). In plants grown upon Mn-deficient conditions, the Fe reductase activity was found to be also induced, although less than in Fe-deficient plants (2.4-fold higher).

### 2.2. Identification of Root Proteins by Shotgun and 2-DE

The LC-MS/MS analysis of all samples detected 1254 proteins in tomato root extracts, and 407 of them were reliably identified and quantified with at least two peptides and therefore considered in this study. The complete list of proteins detected is shown in Table S2, and the raw dataset is available in the ProteomeXchange Consortium via the Pride partner repository with the dataset identifier PXD008326. All these proteins are attributed to the Solanum genus using the ITAG database.

On the other hand, the 2-DE proteomic approach detected 342 consistent spots in extracts from tomato roots, with 49 of them showing statistically significant changes (Student’s *t*-test; *p* ≤ 0.05). When analyzed by nLC-ESI-MS/MS, 88% of these spots (43) were reliably identified, five more spots could not be identified, and in one spot, several proteins were present, and therefore it was not considered in further analyses.

**Figure 2:**
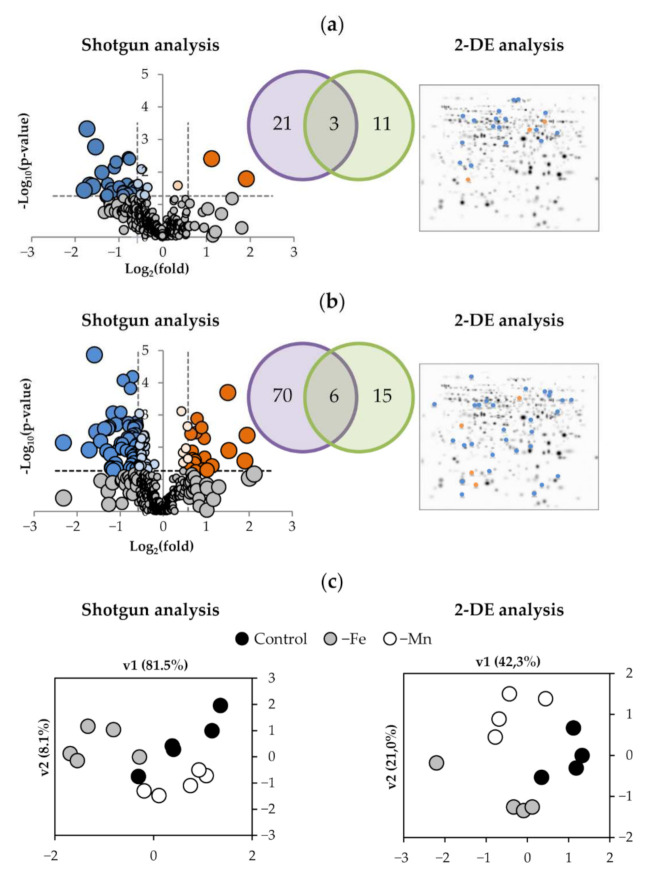
Effect of Fe-deficiency (**a**) and Mn-deficiency (**b**) on the root protein profile and PCA analysis of the data (**c**). The volcano scatter plots show the identified and quantified proteins. Proteins decreasing and increasing in relative abundance (ANOVA, *p* ≤ 0.05) are in blue and orange, respectively, whereas those whose relative abundance was unaffected are in grey. The 2-DE gels shown are master ones, including all the spots detected, with significantly changing spots marked in blue and orange for those decreasing and increasing in relative abundance, respectively. The Venn diagrams show the overlap of differential proteins found by both techniques.

Changes induced by both deficiencies on the root proteome using shotgun analysis are shown in a volcano plot, using the relationship between statistical significance, −log_10_(*p*-value), and biological significance, log_2_(fold-change) (Figure 2a,b). Fe deficiency showed statistically significant (ANOVA; *p* ≤ 0.05) and biologically relevant (fold ≥ 1.50 or fold ≤ 0.67) changes in 24 proteins, whereas Mn deficiency caused changes in 76 proteins. Equivalent samples were analyzed by 2-DE. From the 43 differential spots reliably identified in the 2-DE approach, 14 spots, corresponding to 14 protein species, changed as a result of Fe deficiency, whereas 21 spots, corresponding to 21 protein species, showed changes upon Mn deficiency. All the identified and relevant proteins are presented in Table 2 (for Fe deficiency) and Table 3 (for Mn deficiency). The PCA analysis of the statistically significant changes (ANOVA; *p* ≤ 0.05) measured by 2-DE and shotgun in roots as a result of Fe and Mn deficiencies showed good separation between treatments, with the first and second components explaining approximately 82 and 8% of the variation in the shotgun analysis and 42 and 21 % in the 2-DE analysis (Figure 2c).

### 2.3. Effect of Fe-Deficiency and Mn-Deficiency on the Root Proteome

**Figure 3:**
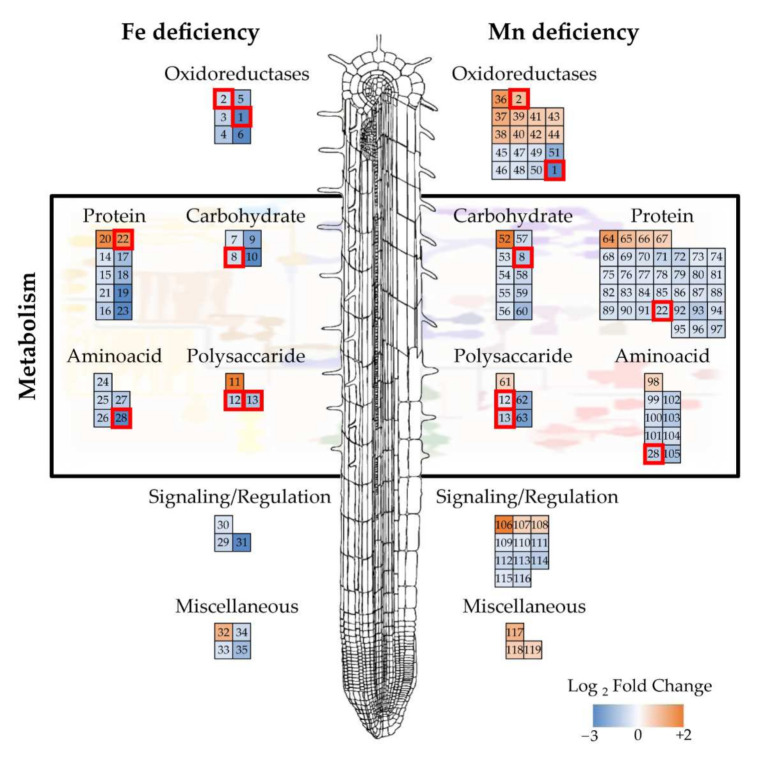
Functional classification and heatmap of proteins accumulated differentially in the root protein profile under Fe deficiency (left panel) and Mn deficiency (right panel). Numbers correspond to those presented in Table 2 and Table 3. Proteins identified to respond to both deficiencies are marked in red squares.

Combining the shotgun and 2-DE datasets, a total of 35 proteins were found to show changes in relative abundance with Fe deficiency. Three of them were detected in both techniques, whereas 21 proteins were detected only by shotgun, and 11 were identified only by 2-DE (Table 2; Figure 2a). Most of them (31) showed significant decreases. Manual functional classification of the proteins decreasing in relative abundance yielded seven functional categories, including oxidoreductases (6 proteins; 17% of the total), carbohydrate process (4 proteins; 11%), polysaccharide metabolism (2 proteins; 6%), protein metabolism (8 proteins; 22%), amino acid metabolism (5 proteins; 14%), signaling/regulation (3 proteins; 9%), and a miscellaneous group (3 proteins; 9%). Regarding those increasing in relative abundance, one, two, and one proteins were in the polysaccharide metabolism, protein metabolism, and miscellaneous categories (3%, 6%, and 3% of the total, respectively). A graphical representation of the Fe deficiency data is shown in Figure 3 (left panel), where the categorized proteins with their corresponding numbers in Table 2 are represented in a heatmap colour-based scheme.

In the case of Mn-deficiency, a total of 91 protein species were found to show changes in relative abundance when the differential proteins obtained by shotgun and 2-DE were combined. Of these, 6 proteins were found using both techniques, whereas 70 proteins were detected only by shotgun and 15 were identified only by 2-DE (Table 3; Figure 2b). From these 91 proteins changing in abundance, Mn deficiency caused decreases in the abundance of 68 proteins. Manual functional classification of these proteins decreasing in abundance yielded six functional categories, including oxidoreductases (8 proteins; 9% of the total), carbohydrate process (9 proteins; 10%), polysaccharide metabolism (4 proteins; 4%), protein metabolism (31 proteins; 34%), and amino acid metabolism (8 proteins; 9%), signaling/regulation (8 proteins; 9%). On the other hand, 23 proteins showed significant increases upon Mn deficiency when compared with control plants. These proteins were manually assigned to seven functional categories: oxidoreductases (10 proteins; 11%), carbohydrate process (1 protein; 1%), polysaccharide metabolism (1 protein; 1%), protein metabolism (4 proteins; 4%), amino acid metabolism (1 protein; 1%), signaling/regulation (3 proteins; 3%), and the miscellaneous group (3 proteins; 3%). A graphical representation of the Mn deficiency data is shown in Figure 3 (right panel), where the categorized proteins with their corresponding numbers in Table 3 are represented in a heatmap color-based scheme.

Only seven proteins changed in abundance as a result of both deficiencies in comparison to control plants as revealed by both techniques (Table 4), and these are highlighted in Figure 3. Five of these proteins showed decreases in both treatments, including two oxidoreductases (proteins 1 and 13), the dTDP-4-dehydro-rhamnose epimerase/reductase (protein 8), the beta-D-xylosidase (protein 12) and the S-adenosylmethionine synthase (protein 28). However, two proteins presented opposite trends between the two treatments: the ribosomal protein L18 (protein 22), which increased in the −Fe roots and decreased in the −Mn ones, and the peroxidase 27 (protein 2), which decreased in the −Fe roots and increased in the −Mn ones.

### 2.4. GO-Enrichment Analysis of Differential Proteins

**Figure 4:**
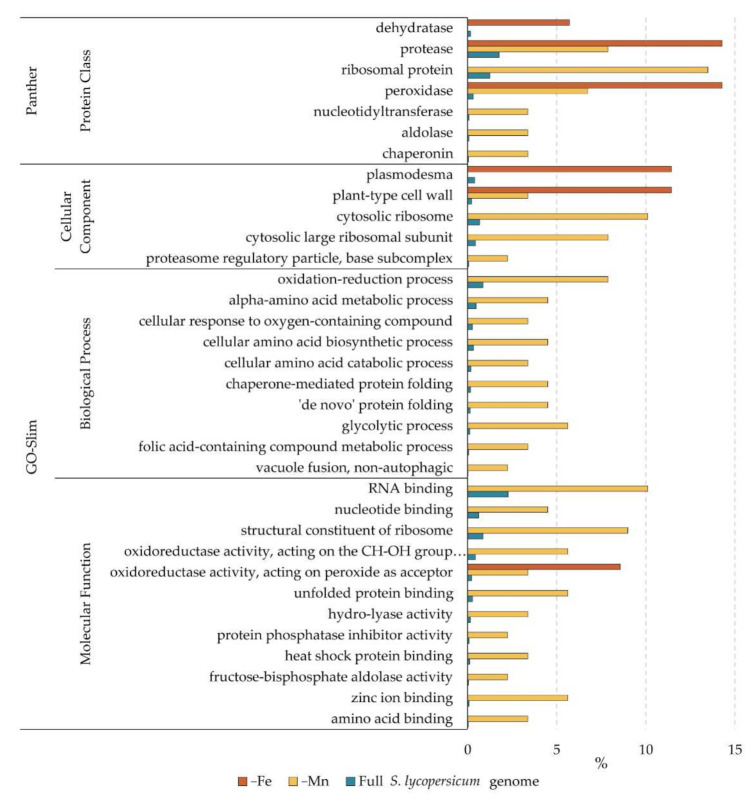
Enrichment in GO categories as a result of Fe and Mn deficiency. Bars represent the percentage of genes of a corresponding category in each dataset (−Fe and −Mn) and in the whole *S. lycopersicum* genome.

The Gene Ontology (GO) knowledgebase is the world’s largest source of information on the functions of genes. The proteins identified to change in abundance under Fe and Mn deficiency in tomato roots were analyzed using the Panther database GO-enrichment tool (www.pantherdb.org, accessed on 10 September 2021). This tool searches for GO functional categories enriched in a given dataset when compared with a reference. The lists of proteins changing as a result of Fe and Mn deficiency were analyzed, using the whole genome annotation of *S. lycopersicum* as reference. Enrichments were calculated using the GO-Slim Molecular Function, Biological Process and Cellular Component databases, as well as the Panther Protein Class database. The complete results are presented in Supporting Material Table S3, and a representation of the enrichment for each category as the percentage of genes present in each dataset and the whole *S. lycopersicum* genome is shown in Figure 4.

Proteins changing under Fe deficiency were enriched for those located to cell wall and plasmodesmata (Figure 4), showing the importance of the extracellular compartment in Fe mobilization and homeostasis. The other category enriched under Fe deficiency included proteins with oxidoreductase activity, specifically peroxidases. Peroxidases can be involved not only in processes related to coping with oxidative stress, well known to occur under Fe deficiency, but also in cell wall modification and synthesis. There was also an enriched number of dehydratases and proteases. The Mn response involved a larger number of proteins changing, and therefore the enrichment analysis gave more hits (Figure 4). In addition to the categories plant-type cell wall and peroxidases, protein-related processes (such as ribosomal related compartments, proteasome, heat shock and unfolded protein binding, and chaperonins) were found to be over-represented in the dataset. In addition to the peroxidases present in the Fe deficiency dataset, a different category of oxidoreductases (those acting on the CH-OH group as acceptor) was also over-represented. To a lesser extent, there were enrichments calculated for RNA, nucleotide, and metal-binding proteins, as well as proteins involved in general and amino acid metabolism.

## 3. Discussion

Iron and manganese deficiencies caused leaf chlorosis and photosynthetic pigment changes consistent with those found in previous studies with tomato plants [41]. Changes in metal concentrations in roots are also indicative of typical Fe and Mn deficiencies.

### 3.1. The Cell Wall Is a Key Component to Fe and Mn Deficiencies

According to the results, both metal deficiencies caused a major reorganization of the cell wall. Changes were observed in proteins related to polysaccharide metabolism, specifically on those related to the cell wall (Table 2 and Table 3, proteins 11–13 and 61–63). Furthermore, the cell wall compartment was relatively enriched in both protein datasets (Figure 4). The cell wall has been described before as a major sink for metals, in fact constituting a metal accumulation tissue. Under heavy metal toxicity, cell walls increase in size in order to retain the metal outside the cells in a blocking strategy [42,43]. Lately, specific cell wall modifications have been suggested to be involved in metal sequestration, and therefore this may constitute an important trait in the metal deficiency responses [44]. Under Fe deficiency, decreases in two proteins related to cell wall degradation (proteins 8 and 12) and cell wall monomer biosynthesis (protein 13), and an increase in an apyrase (protein 11), whose closest homolog is a beta-D-glucosidase (Table 2, Figure 3) were observed. These changes point towards a remodeling of the cell wall under Fe deficiency. Changes observed under Mn deficiency were even more marked, and in addition to the two proteins decreasing under Fe deficiency (Table 4 and Figure 3, proteins 8 and 12), a protein involved in cell wall degradation (protein 62) and another one involved in cell wall modification (protein 63) were found to be decreased (Table 3, Figure 3). Interestingly, a pectinesterase (protein 61, Figure 3) was found to increase in relative abundance upon Mn deficiency. This family of enzymes remove methyl groups from pectin, leaving behind hydroxyl groups which may be able to chelate and fix divalent metals [45].

### 3.2. Contrasting Changes in Protein Profiles with Fe and Mn Deficiencies

Both Fe and Mn are essential micronutrients for plants, but they have been shown to elicit different, and sometimes opposite, responses when they are scarce in the growth medium [32,35,36]. In the results shown here, this antagonism is reflected in contrasting changes in oxidoreductase enzymes, coumarin production, and protein and energy metabolism.

#### 3.2.1. Oxidoreductases and Oxidative Stress Responses

Both mineral deficiencies cause a major change in proteins with oxidoreductase functions, especially regarding peroxidase enzymes (Figure 4). Iron deficiency caused a major decrease in up to six different peroxidases (Table 2 and Figure 3, proteins 1–6). Contrasting observations regarding oxidoreductase responses under Fe deficiency have been reported [46,47,48,49]. Literature suggests that increases in antioxidant enzymes under Fe deficiency in response to oxidative stress is more intense in cultivars sensitive to Fe deficiency than in tolerant cultivars [50,51]. The decreases observed in this study suggest that tomato is well adapted and tolerant to Fe deficiency. Perhaps, as peroxidases have a Fe-S center in their reactive site, in case of Fe scarcity, this family of proteins are sacrificed to use the available Fe towards other higher priority functions, including energy production and photosynthesis. Under Mn deficiency, the situation is different since there is an increase in the Fe available in the root (Figure 1), which probably leads to intracellular redox stress. This extra pool of Fe is reflected in the increase in oxidoreductase enzymes observed (Table 3 and Figure 3, proteins 36–44). It is important to note that some of these increases occur not only among peroxidases but also in enzymes devoted to coping with oxidative stress, such as a thioredoxin reductase (protein 37), a monodehydroascorbate reductase (protein 38), and an alcohol dehydrogenase (protein 41).

#### 3.2.2. Coumarin Production

Catechol-containing coumarin-type compound production is a major trait of some plant species under Fe deficiency. This issue has been extensively studied in the Fe homeostasis field in the last decade [24,25,26,52]. Surprisingly, no protein related to this pathway was found in the Fe deficiency dataset (Table 2). However, three proteins potentially related to this pathway appear in the Mn deficiency dataset (Table 3). Two proteins from the 2-oxoglutarate dioxygenase family were decreased under Mn deficiency (proteins 45 and 51). This family of proteins has been shown to be involved in key steps of the production of catechol coumarins [26,52]. Interestingly, a third protein, a polyphenol oxidase (protein 44), was found to be increased under Mn deficiency. This protein has also been described as a catechol oxidase, catalyzing the oxidation of di-phenols to di-quinones, thus destroying the metal-chelation capabilities of this type of compound [53]. These data suggest that under Mn deficiency, not only the production of catechol coumarins is decreased, but also any potential catechol coumarin present is deactivated, potentially as a way of reducing their Fe uptake. Indeed, a catechol oxidase was found to be decreased in tomato roots under Fe deficiency, further confirming this hypothesis [48].

#### 3.2.3. Protein and N Metabolism

Reorganization of the protein metabolism has been observed both for Fe deficiency [46] and for Mn deficiency [32]. In the present study, both metal deficiencies provoked marked changes in protein metabolism (Table 2 and Table 3, proteins 14–23 and 64–97), and this fact is reflected in enrichments in the protease, chaperonin, ribosome, proteasome, and amino acid-related categories (Figure 4). However, when looking at individual protein changes, the response to both metal deficiencies is different. Under Fe deficiency, we observe a relative abundance decrease in six proteases (Table 2, proteins 14–19), with increases observed for a chaperone (protein 20) and a ribosomal protein (protein 22). All proteins related to amino acid metabolism were found to be decreased (Table 2, proteins 24–28). This suggests that under Fe deficiency, to cope with a decreased N uptake (whose machinery needs Fe), the proteins already synthesized are tried to be conserved via decreases in degradation and help in protein folding. De novo protein synthesis seems to be favoured, but not from newly synthesized amino acids. Increases in chaperones under Fe deficiency has been already described in tomato [48], whereas in *Medicago truncatula*, proteases were increased instead [46]. Under Mn deficiency, a general shutdown of proteins related to protein synthesis (proteins 80–94), folding (proteins 73–79), and amino acid biosynthesis (proteins 99–105) was observed (Table 3 and Figure 3). The case of proteases was more complex (Table 3), with some being increased (proteins 64–67), and some decreased (proteins 68–72), as opposed to the general decrease observed under Fe deficiency (Table 2, proteins 14–19). It is interesting to note that the large number (up to 13) of ribosomal proteins decreased in abundance under Mn deficiency (Table 3). This ribosomal reorganization was observed transcriptionally under Fe deficiency [54], and translational control was suggested to be of great importance in the Mn deficiency response [32].

#### 3.2.4. Different Ways to Cope with a Common Problem: The Lack of Energy

Both Fe and Mn are essential for the energy-producing machinery of plants, although at different points. Iron is essential as a cofactor of nearly all the proteins involved in electron transfer machinery, especially in mitochondria, whereas Mn is essential for the water-splitting complex, the key component of photosynthesis. Probably due to this difference, the way the roots of tomato seem to cope with the lack of energy differs. Under Fe deficiency, a general decrease is observed in carbohydrate metabolism (Table 2 and Figure 3, proteins 7–10). These data contrast the previously found increases in carbohydrate metabolism and TCA cycle under Fe deficiency [46,48]. Interestingly, a mitochondrial processing peptidase (protein 23) and a mitochondrial ATP synthase (protein 35) were also decreased, pointing towards a shutdown of mitochondrial respiratory ATP production, in line with suggested increases in fermentation found in the literature [46,47,48]. Under Mn deficiency, we observed a similar decrease in carbohydrate metabolism (proteins 53–60) but also increases in two subunits of ATP synthase (proteins 117 and 119). This could be due to a lack of energy coming from photosynthesis and a mechanism to cope with it by increasing the mitochondrial machinery. This would be in line with previous results in *Arabidopsis*, where strong decreases in glycolysis and TCA cycle in coordination with decreases in fermentative enzymes pointed towards a preference for respiration in energy production [32].

### 3.3. A Possible Role of Extracellular Proteins in the Fe Deficiency Response

Interestingly, we observed changes in several proteins under Fe deficiency which could function in the intercellular space. This is the case of two germins (Table 2, proteins 32 and 34), putatively binding Mn and located to plasmodesmata, which explains the fact that this compartment is over-represented in the dataset (Figure 3). Other proteins supposed to be present at the cellular surface found to be changing are a hormone receptor (protein 29) and a remorin (protein 33), a protein putatively binding Fe(II) (Table 2). However, the involvement of these proteins in the Fe deficiency response remains unclear and they will deserve further studies in the future.

### 3.4. Mn Deficiency and Pathogen Response

An increase in proteins related to pathogen response is observed under Mn deficiency (Table 3, proteins 106–108). Changes in processes related to pathogen response have been described before in relation to Mn deficiency, as is the case of an increase observed in glucosinolate biosynthesis in Arabidopsis [32]. The fact that glucosinolates are a metabolite class specific to Brassicaceae would explain why, in tomato, pathogenesis-related proteins are elicited instead. In wheat, a decrease was observed in lignin concentration from Mn-deficiency plants, specifically in roots, which also made them more prone to fungal diseases and, in combination with the reduced biomass, less able to compete again weed species [55].

## 4. Materials and Methods

### 4.1. Plant Material and Sampling

Tomato (*Solanum lycopersicum* L., cv. Tres Cantos) plants were grown hydroponically in a controlled environment chamber (Fitoclima 10,000 EHHF, Aralab, Lisbon, Portugal) with a photosynthetic photon flux density (PPFD) of 400 µmol m^−2^ s^−1^ photosynthetically active radiation at the leaf level, 80% relative humidity, and a photoperiod of 16 h, 23 °C/8 h, 18 °C day/night regime. In the Fe deficiency experiment, seeds were germinated in vermiculite for 13 days in a half-strength Hoagland nutrient solution containing 45 µM Fe-EDTA and 4.6 µM MnCl_2_. Seedlings were then transplanted to 10 L plastic buckets (16–18 plants per bucket) containing a half-strength Hoagland nutrient solution and grown for an additional 13-day period. After this time, solutions were renewed, and control (45 µM Fe (III)-EDTA, 4.6 µM MnCl_2_) and Fe-deficiency treatments (0 µM Fe (III)-EDTA, 4.6 µM MnCl_2_) were imposed for 8 more days. In the Mn experiment, the timeline and experiment design (including control conditions) were the same, but Mn-deficient plants (0 µM MnCl_2_) were germinated and grown without Mn and with 45 µM Fe (III)-EDTA throughout the experiment, collecting the samples from 34-day-old plants. Roots and leaf disks were collected at the end of each treatment, frozen in liquid N_2_, and kept at −20 °C until analysis. A graphical representation of the plant material production is presented in Supplementary Figure S1.

### 4.2. Experimental Design

One independent experiment consisted of 2 buckets (one bucket per treatment) containing 16–18 plants each, with roots from 2 plants per bucket being pooled, used for protein extraction, and considered as a biological replicate. This setup was repeated 4 times for the 2-DE electrophoresis analysis (*n* = 4) and 5 times for the shotgun analysis (*n* = 5).

For mineral analysis, roots from 2 plants per treatment were sampled separately and mean values calculated, and the experiment was repeated 5 times (*n* = 5). Photosynthetic pigment analyses were carried out in 3 plants per treatment (using 5 disks from 2 leaves in each plant) in at least 5 independent experiments (*n* = 15). For Fe reductase activity, measurements were carried out in 8 plants per treatment in a single experiment (*n* = 8).

### 4.3. Mineral Analysis, Chlorophyll Estimation and Photosynthetic Pigment Analysis

For nutrient analysis, roots, stems, and leaves were sampled, washed, dried, and milled using standard procedures [41]. Plant tissues (100 mg dry weight) were digested in a microwave system (Milestone Ethos Plus, Bergamo, Italy) with 6.4 mL HNO_3_ (26%, TraceSelect Ultra, Sigma-Aldrich, Madrid, Spain) and 1.6 mL H_2_O_2_ (30%). The concentrations of the micro (Fe, Mn, Cu and Zn) and macronutrients (Ca, Mg and K) were determined by flame atomic absorption (AAS) and flame emission (FES; only for K) spectrometry using a Solaar 969 apparatus (Unicam Ltd., Cambridge, United Kingdom).

Leaf chlorophyll content was estimated using a SPAD 502 apparatus (Minolta Co., Osaka, Japan). SPAD values of young and old expanded leaves were recorded at sampling time (8 days after treatment onset), and an average of the measurements per treatment was obtained (4 leaves per treatment and 4 measurements per leaf).

For pigment analysis, leaves were sampled from 2 leaf levels, young and old ones, as indicated in detail in a previous paper [41]. Leaf disks were sampled using a calibrated 0.5-cm diameter cork-borer 8 days after treatment onset, wrapped in aluminum (Al) foil, frozen in liquid N_2_, and stored at −20 °C until analysis. Leaf pigments were extracted with acetone in the presence of Na ascorbate and stored following the procedure described in [56]. Pigment extracts were thawed on ice, filtered through a 0.22-μm polytetrafluoroethylene PTFE filter, and analyzed by high-performance liquid chromatography using a Waters 600 pump and 996 photodiode array detector (Waters Co., Milford, MA, USA) as in [57]. The total concentrations of Chl (Chl a and Chl b), neoxanthin, violaxanthin (V), lutein epoxide (taraxanthin), antheraxanthin (A), lutein, and zeaxanthin (Z) were measured. The ratios Chl a/Chl b and (A + Z)/(V + A + Z) were also calculated.

### 4.4. Root Iron Reductase Activity

The root ferric reductase activity (FCR) of intact, illuminated plants was determined by following the formation of the Fe(II)-BPDS complex from Fe(III)-EDTA [58]. Eight days after the treatment onset, individual plants were transferred to 250 mL beakers containing 5 mM Mes-KOH pH 5.5 solution supplemented with 300 µM BPDS and 500 µM Fe(III)-EDTA. The beaker was fully covered with Al foil, and the solution was aerated continuously. Aliquots were collected 30 min after placing plants in the beakers, and absorbance was measured at 535 nm with a spectrophotometer (UV2450, Shimadzu, Kyoto, Japan). An extinction coefficient of 22.14 mM^−1^ cm^−1^ was used for the estimation of reduced Fe. Blanks were made in the absence of plants to correct for any photoreduction and also in the presence of plants but adding Fe only at the end of the reaction to correct for the Fe reduction caused by secreted substances.

### 4.5. Protein Extraction

Root material (approximately 0.5–1.0 g, pooled from 2 plants of a given treatment) was ground in liquid N_2_ with mortar and pestle, and then homogenized in 6 mL of phenol saturated with Tris-HCl 0.1 M (pH 8.0) containing 5 mM β-mercaptoethanol, by stirring for 30 min at 4 °C. After incubation, the homogenate was filtered (PVDF, 0.45 μm) and centrifuged at 5000× *g* for 15 min. The phenol phase was re-extracted for 30 min with one volume of phenol saturated Tris-HCl 0.1 M (pH 8.0) containing 5 mM β-mercaptoethanol, and centrifuged as described above. The phenol phase was collected, and proteins precipitated by adding 5 volumes of cold 0.1 M ammonium acetate in methanol. Samples were kept overnight at −20 °C and centrifuged at 20,000× *g* for 20 min. The pellet was washed twice with cold methanol, dried with N_2_ gas, and solubilized in a sample rehydration buffer containing 8 M urea, 2% (*w*/*v*) CHAPS, 50 mM DTT, 2 mM PMSF, and 0.2% (*v*/*v*) IPG buffer pH 3–10 (GE Healthcare, Uppsala, Sweden). After rehydration, samples were incubated in a Thermomixer Comfort device (Eppendorf AG, Hamburg, Germany) at 42 °C and 1000 rpm during 3 h, centrifuged at 10,000× *g* for 10 min at RT, and filtered (0.45 µm ultrafree-MC filters, Millipore, Bedford, MA, USA). Protein concentration was quantified immediately with the Bradford (Sigma-Aldrich, St. Louis, MO, USA) method using microtiter plate spectrophotometer (Asys UVM 340, Biochrom Ltd., Cambridge, United Kingdom) and BSA as standard.

### 4.6. Label-Free Liquid Chromatography-Tandem Mass Spectrometry (LC-MS/MS)

Sample preparation for label-free LC-MS/MS was carried out according to [59] and [60]. Briefly, 5 µg of total proteins were subjected to 1-DE to remove nonprotein compounds, the resulting gel bands were cut into six pieces, proteins were in-gel digested with trypsin, and the resultant peptides were purified by using a solid-phase extraction tip (AMR, Tokyo, Japan).

For peptide separation, an ADVANCE UHPLC system (Michrom Bioresources, Auburn, CA, USA) was used [61]. Peptide solutions were concentrated in a trap column (Lcolumn Micro 0.3 × 5 mm; CERI, Japan), elution was carried out with 0.1% (*v*/*v*) formic acid in ACN, and peptides were separated in a Magic C18 AQ nano column (0.1 × 150 mm; Michrom Bioresources) with a linear gradient of ACN (from 5% to 45%) and a flow rate of 500 nL min^−1^. Mass spectrometry analysis was carried out on an LTQ Orbitrap XL device (Thermo Fisher Scientific, Waltham, MA, USA), carrying out peptide ionization with a spray voltage of 1.8 kV and an ADVANCE spray source (Michrom Bioresources). Data acquisition parameters were set, as in [62], and Xcalibur v. 2.0.7 (Thermo Fisher Scientific) was used as instrument control software.

Mass data analysis was performed as described previously [40,60,61]. Protein identification was carried out using the full peptide list with the Mascot search engine (version 2.4.1, Matrix Science, London, UK) and the ITAG v4.0 database 20190914 (34,075 sequences). Search parameters were: peptide mass tolerance ± 5 ppm, MS/MS tolerance ± 0.6 Da, one allowed missed cleavage, allowed fixed modification carbamidomethylation (Cys), and variable modification oxidation (Met) and peptide charges were set to +1, +2 and +3. Positive protein identification was assigned with at least two unique top-ranking peptides with scores above the threshold level (*p* ≤ 0.05). Protein information was exported from Mascot .xml format and imported to Progenesis QI for proteomics software (v. 2.0, Nonlinear Dynamics, Newcastle upon Tyne, United Kingdom), which associates peptide and protein information. The MS proteomics data have been deposited to the ProteomeXchange Consortium via the [63] partner repository, with the data set identifier PXD008326.

To assess the effect of Fe and Mn deficiencies in the protein profile of tomato roots, we calculated the ratio of normalized protein abundance between metal deficient and control samples. Only changes with a *p* ≤ 0.05 (ANOVA) and a ratio ≥1.50 or ≤0.67 were considered statistically significant and biologically relevant, respectively. Multivariate statistical analyses (Principal Component Analysis; PCA) were carried out using SPSS Statistical software (v. 24.0), including only proteins showing statistically significant changes (ANOVA; *p* ≤ 0.05) as a result of the Fe- and Mn-deficient treatments.

The GO biological process annotation (http://www.geneontology.org, accessed on 10 September 2021) and domain annotations were used for classification of each individual protein identified into 9 different functional categories as follows: defence, oxido-reductases, protein metabolism, carbohydrate metabolism, polysaccharide metabolism, signaling, lipid metabolism, amino acid metabolism, acid nucleic metabolism, and a miscellaneous group containing other functional categories.

### 4.7. Protein 2-DE Separation of Root Samples

Preliminary 2-DE experiments were carried out in root samples using a first dimension IEF separation on 7 cm ReadyStrip IPG Strips (BioRad, Hercules, CA, USA) with a linear pH gradient pH 5–8 using a Protean IEF Cell (BioRad). Strips were rehydrated for 16 h at 20 °C in 125 μL of rehydration buffer containing 80 μg protein and a trace of bromophenol blue and then transferred onto a strip tray. IEF was run at 20 °C for a total of 14000 V h (20 min with a 0–250 V linear gradient, 2 h with a 250–4000 V linear gradient and 4000 V until 10 000 V h). After IEF, strips were equilibrated for 15 min in equilibration solution I [6 M urea, 0.375 M Tris-HCl, pH 8.8, 2% (*w*/*v*) SDS, 20% (*v*/*v*) glycerol, 2% (*w*/*v*) DTT] and for another 15 min in equilibration solution II [6 M urea, 0.375 M Tris-HCl pH 8.8, 2% (*w*/*v*) SDS, 20% (*v*/*v*) glycerol, 2.5% (*w*/*v*) iodoacetamide]. For the second dimension SDS-PAGE, equilibrated IPG strips were placed on top of vertical 12% SDS-polyacrylamide gels (8 × 10 × 0.1 cm) and sealed with melted 0.5% agarose in 50 mM Tris HCl, pH 6.8, containing 0.1% SDS. SDS-PAGE was carried out at 20 mA per gel for approximately 1.5 h, until the bromophenol blue reached the plate bottom, in a buffer containing 25 mM Tris, 192 mM glycine, and 0.1% SDS, at 4 °C. Gels were subsequently stained with Coomassie-blue R-250 (Sigma-Aldrich, St. Louis, MO, USA).

### 4.8. Gel Image and Statistical Analysis

Stained gels were scanned with an Epson Perfection 4990 Photo Scanner (Epson Ibérica, Barcelona, Spain) at 600 dpi, previously calibrated using the SilverFast 8 software (LaserSoft Imaging AG, Kiel, Germany) and an IT8 reference card. Spot detection, gel matching and interclass analysis were performed with PDQuest 8.0 software (BioRad). First, normalized spot volumes based on the total intensity of valid spots were calculated for each 2-DE gel and used for statistical calculations of protein abundance; for all spots present in the gels, pI, Mr, and normalized volumes (mean values and SD) were determined. Experimental Mr values were calculated by mobility comparisons with Precision Plus protein standard markers (BioRad) run in a separate lane on the SDS gel, and pI was determined using a linear scale over the total dimension of the IPG strips. Only spots consistently present in 100% of the replicates (four gels) from at least one class were considered and used in further analysis. The spots were also manually checked, and consistent reproducibility between normalized spot volumes was found in the different replicates.

Spots changing in relative abundance were selected using a paired Student’s *t*-test and a significance level of *p* ≤ 0.05. Protein response ratios were defined as the relative abundance in the treatment divided by the relative abundance in the control.

### 4.9. Protein in Gel Digestion

Spots were excised automatically using an EXQuest spot cutter (BioRad), transferred to 500 μL Protein LoBind Eppendorf tubes, destained in 400 μL of 40% [*v*/*v*] acetonitrile (ACN) and 60% [*v*/*v*] 200 mM NH_4_HCO_3_ for 30 min and dehydrated in 100% ACN for 10 min. Gel pieces were dried at room temperature and then in-gel digested with 15 μL Trypsin solution (Sequencing grade Modified Trypsin V511, Promega, Madison, WI, USA; 0.1 μg μL^−1^ in 40 mM NH_4_HCO_3_/9% ACN). After incubation o/*n* at 37 °C, the reaction was stopped by adding 1 μL of 1% TFA. The peptide solution was finally analyzed using MS.

### 4.10. Protein Identification by Nanoliquid Chromatography-Tandem Mass Spectrometry (nLC−ESI−MS/MS)

Peptides present in 6 μL of the sample were preconcentrated on line onto a 300 μm i.d. × 5 mm, 5 μm particle size ZORBAX 300SB-C18 trap column (Agilent Technologies, Waldbronn, Germany), using a 100 μL min^−1^ flow rate of 3% ACN, 0.1% formic acid, in a nano-HPLC system 1200 series (Agilent Technologies). Backflow elution of peptides from the trap column was carried out, and separation was done with a 75 μm i.d. × 150 mm, 3.5 μm particle size ZORBAX 300SB-C18 column (Agilent Technologies), using a 300 nL min^−1^ nanoflow rate and a 55 min linear gradient from solution 97% A (0.1% formic acid) to 90% of solution B (90% ACN, 0.1% formic acid). The nano-HPLC was connected to an HCT Ultrahigh-capacity ion trap (Bruker Daltoniks, Bremen, Germany) using a PicoTip emitter (50 μm i.d., 8 μm tip i.d., New Objective, Woburn, MA, USA) and an online nanoelectrospray source. The capillary voltage was −1.8 kV in positive mode, and a dry gas flow rate of 10 L min^−1^ was used at 180 °C. The scan range used was from 300 to 1500 m/z. The mass window for precursor ion selection was ±0.2 Da, and the rest of the parameters were those recommended by the manufacturer for MS/MS proteomics work. Peak detection, deconvolution and processing were performed with Data Analysis 3.4 software (Bruker Daltoniks, Bremen, Germany).

Protein identification was carried out using the Mascot search engine (Matrix Science; London, United Kingdom) and the non-redundant databases ITAG v4.0 20190914 (34,075 sequences). Search parameters were: monoisotopic mass accuracy, peptide mass tolerance ±0.3 Da, fragment mass tolerance ±0.6 Da; one allowed missed cleavage; allowed fixed modification carbamidomethylation (Cys), and variable modification oxidation (Met). Positive identification was assigned with Mascot scores above the threshold level (*p* ≤ 0.05), at least 2 identified peptides with a score above homology and similar experimental and theoretical molecular weight and pI values. We used the GO biological process annotation (http://www.geneontology.org, accessed on 10 September 2021) and domain annotations for protein classification.

## 5. Conclusions

The present work highlights the interplay between Fe and Mn homeostasis. Data presented show common, opposite, and specific responses to both deficiencies. Cell wall remodeling and modification seems to be key aspect to both metal deficiencies. Further work deserves to be aimed at studying the function of this understudied compartment that serves as a communication platform between the rhizosphere and the roots, and between cells, for metal uptake and trafficking. Data presented show that the response to Fe and Mn deficiency differs in relation to oxidoreductase proteins, coumarin biosynthesis, and protein metabolism. Oxidoreductase enzymes appear to be key players in response to the stress produced by metal deficiencies and toxicities. How this response is regulated and the influence of the quantity, quality, and activity of these enzymes in the susceptibility of each plant cultivar to a given metal deficiency deserves further attention. Protein metabolism and regulation, including synthesis, degradation, and folding, arise as another field of study in metal homeostasis. Post-translational regulation of proteins is of key importance to plants response to the environment. Changes observed in ribosomal proteins could point towards a tailored ribosomal composition which could affect the set of proteins translated under a set of circumstances, providing a new regulatory level yet to be explored.

Future works will need to explore the interactions between both deficiencies at the regulatory level in order to find the regulation hubs governing the responses observed in the present work and others.

## Figures and Tables

**Table 1:** SPAD values, concentrations of photosynthetic pigments (in μmol m^−2^) and pigment ratios in young, expanded leaves of tomato plants grown in control, Fe deficiency, and Mn deficiency conditions. Data are means ± SE (*n* = 15 plants). Different letters in the same row indicate statistically significant differences (Student’s *t*-test, *p* ≤ 0.05).

	Control	-Fe	-Mn
SPAD	42.4 ± 0.7 a	13.6 ± 0.5 b	29.6 ± 0.8 c
Total Chl (Chl a + Chl b)	323.9 ± 14.1 a	88.0 ± 13.5 b	219.5 ± 11.3 c
Neoxanthin	12.0 ± 0.6 a	2.4 ± 0.2 b	6.7 ± 0.4 c
Violaxanthin (V)	17.6 ± 0.9 a	4.6 ± 0.5 b	10.0 ± 0.7 c
Taraxanthin	0.2 ± 0.1 a	0 a	1.6 ± 0.2 b
Antheraxanthin (A)	0.8 ± 0.2 a	1.9 ± 0.1 b	1.3 ± 0.2 c
Lutein	44.8 ± 1.6 a	15.3 ± 0.7 b	24.5 ± 1.3 c
Zeaxanthin (Z)	0.2 ± 0.1 a	3.1 ± 0.4 b	0.3 ± 0.1 a
(V + A + Z)	18.6 ± 0.8 a	9.5 ± 0.4 b	11.5 ± 0.6 c
Chl a/Chl b ratio	3.0 ± 0.1 a	2.8 ± 0.1 a	3.0 ± 0.1 a
(A + Z)/(V + A + Z) ratio	0.06 ± 0.01 a	0.53 ± 0.04 b	0.14 ± 0.03 c

**Table 2:** Identified and quantified proteins (35 in total) changing in relative abundance by Fe deficiency. Accession code is from the ITAG 4.0 database. Fold SG—fold change found between Fe-deficient and control samples by shotgun in a log_2_ base. Fold 2D—fold change found between Fe deficient and control samples by 2-DE. Relative abundance increases are underlined for the sake of clarity. Mn—response observed in Mn-deficient samples compared to controls (− decreased abundance, + increased abundance).

#	Accession	UniProtKB	Description	Fold SG	Fold 2D	Mn
Oxidoreductases (6)						
1	Solyc07g052510.4.1	A0A3Q7HDZ4	peroxidase (TPX1)	−0.76	−3.00	-
2	Solyc12g005790.2.1	A0A3Q7JR84	peroxidase 27	−0.82		+
3	Solyc04g071890.3.1	A0A3Q7G7T0 *	peroxidase	−1.06		
4	Solyc10g076245.1.1	A0A3Q7IJN4 *	peroxidase 70	−1.40		
5	Solyc05g046000.4.1	A0A3Q7GKW8	peroxidase 27-like	−1.73		
6	Solyc10g076210.2.1	A0A3Q7IJN4 *	peroxidase 1		−3.00	
**Carbohydrate Metabolism (4)**						
7	Solyc05g050800.3.1	A0A3Q7GLU0	phosphoglycerate mutase family	−0.65		
8	Solyc08g080140.4.1	A0A3Q7HT77	bifunctional dTDP-4-dehydrorhamnose 3,5-epimerase/reductase	−0.81		-
9	Solyc07g052350.3.1	A0A3Q7HBK4	aconitate hydratase	−1.17	−3.00	
10	Solyc06g073190.3.1	Q42896	fructokinase-like		−2.30	
**Polysaccharide Metabolism (3)**						
11	Solyc12g098540.2.1	A0A3Q7JEE6 *	apyrase	1.91		
12	Solyc01g104950.4.1	A0A3Q7EQN7	beta-D-xylosidase 2 precursor	−0.90	−1.14	-
13	Solyc01g107590.3.1	A0A3Q7ESC5	cinnamyl alcohol dehydrogenase	−1.54		-
**Protein Metabolism (10)**						
14	Solyc08g067100.2.1	K4CLT6	eukaryotic aspartyl protease family	−0.61		
15	Solyc05g013820.4.1	A0A3Q7GHJ1	proteasome subunit beta type-7-A	−0.73		-
16	Solyc08g079920.2.1	A0A3Q7HVI4 *	P69f protein	−1.16		
17	Solyc03g019690.1.1	A0A3Q7FGU5	Kunitz-type protease inhibitor	−1.60		
18	Solyc08g079930.2.1	A0A3Q7HVI4 *	subtilisin-like protease	−1.79		
19	Solyc02g081700.1.1	A0A3Q7F6F6	proteasome subunit alpha type		−3.00	
20	Solyc08g082820.4.1	A0A3Q7HX02	glucose-regulated protein 78		1.69	
21	Solyc01g028810.3.1	A0A3Q7ECG0	chaperonin		−0.75	
22	Solyc01g099900.4.1	A0A3Q7ENE9	ribosomal protein L18		1.51	
23	Solyc12g008630.2.1	A0A3Q7J3G4	mitochondrial processing peptidase alpha subunit		−3.00	
**Aminoacid Metabolism (5)**						
24	Solyc01g080280.3.1	A0A3Q7EI59	chloroplast glutamine synthetase	−0.79		
25	Solyc06g060790.1.1	A0A3Q7GXH5	3-isopropylmalate dehydratase	−1.11		
26	Solyc11g011380.2.1	A0A3Q7IRF8 *	glutamine synthetase	−1.26		
27	Solyc12g005080.2.1	A0A3Q7J1A5	dihydrolipoyllysine-residue succinyltransferase component of 2-oxoglutarate dehydrogenase		−0.84	
28	Solyc09g008280.2.1	P43282	S-adenosylmethionine synthase		−3.00	-
**Signaling/Regulation (3)**						
29	Solyc08g076960.1.1	A0A3Q7HTY9	abscisic acid receptor PYL1	−1.00		
30	Solyc07g062110.3.1	A0A3Q7IAM5	protein FLX-like 1		−0.59	
31	Solyc02g093340.3.1	A0A3Q7FDP2	heterogeneous nuclear ribonucleoprotein A3		−3.00	
**Miscellaneous (4)**						
32	Solyc01g102390.4.1	A0A3Q7EP74 *	germin-like protein 5-1	1.12		
33	Solyc03g025850.3.1	Q9XEX8	remorin 1	−0.66		
34	Solyc03g113570.1.1	A0A3Q7FQV4 *	germin-like protein	−0.91		
35	Solyc05g008460.4.1	A0A3Q7GD18	ATP synthase subunit beta	−1.66		

* When the accession sequence gave no hit in the Uniprot database, the closest homolog found in *Solanum lycopersicum* was used.

**Table 3:** Identified and quantified proteins (91 in total) changing in relative abundance by Mn deficiency. Accession code is from the ITAG 4.0 database. Fold SG—fold change found between Mn deficient and control samples by shotgun in a log_2_ base. Fold 2D—fold change found between Mn deficient and control samples by 2-DE. Relative abundance increases are underlined for the sake of clarity. Fe—response observed in Fe-deficient samples compared to controls (− decreased abundance, + increased abundance).

#	Accession	UniProtKB	Description	Fold SG	Fold 2D	Fe
Oxidoreductases (18)						
36	Solyc02g084780.4.1	A0A3Q7F7C4 *	peroxidase superfamily protein	1.53		
37	Solyc03g032000.4.1	A0A3Q7G4N1	thioredoxin reductase 2-like		1.21	
38	Solyc08g081530.3.1	A0A3Q7HU15	monodehydroascorbate reductase	0.96		
2	Solyc12g005790.2.1	A0A3Q7JR84	peroxidase 27	0.93		-
39	Solyc02g062510.3.1	A0A3Q7F0H1	peroxidase	0.89		
40	Solyc10g076240.3.1	A0A3Q7IJN4	cationic peroxidase 1	0.79		
41	Solyc05g056540.4.1	A0A3Q7HI60	alcohol dehydrogenase 1B	0.76		
42	Solyc02g084800.4.1	A0A3Q7F7C4	peroxidase 72	0.73		
43	Solyc10g050890.2.1	A0A3Q7IH89	nitrite reductase 2	0.68		
44	Solyc02g078650.4.1	A0A3Q7F625	polyphenol oxidase	0.62		
45	Solyc11g072550.2.1	A0A3Q7J0V5	4,5-dioxygenase-like protein	−0.63		
46	Solyc06g005150.3.1	Q52QQ4	ascorbate peroxidase	−0.64		
47	Solyc01g100360.4.1	A0A3Q7ENY7	dihydrolipoyl dehydrogenase 2	−0.68		
48	Solyc09g007520.3.1	A0A3Q7HVX4	peroxidase	−0.78	−0.74	
49	Solyc09g011240.3.1	A0A3Q7I069	aldo-keto reductase 4B-like	−0.84		
50	Solyc06g059740.4.1	A0A3Q7GTE7	alcohol dehydrogenase 2	−1.10		
51	Solyc07g043420.3.1	Q40131	2-oxoglutarate-dependent dioxygenase 2	−2.32		
1	Solyc07g052510.4.1	A0A3Q7HDZ4	peroxidase		−3.64	-
**Carbohydrate Metabolism (10)**						
52	Solyc05g005490.4.1	Q5NE21	carbonic anh isoform 1	1.95		
53	Solyc03g115990.3.1	A0A3Q7GH43	malate dehydrogenase	−0.66		
54	Solyc09g075450.3.1	A0A3Q7I742	fumarate hydratase	−0.69		
55	Solyc09g009260.3.1	A0A3Q7HX95	fructose-1,6-bisphosphate aldolase	−0.71		
56	Solyc04g011400.3.1	A0A3Q7FZG5	UDP-glucuronate decarboxylase 1	−0.71		
57	Solyc10g083570.3.1	A0A3Q7ILY0	fructose-bisphosphate aldolase	−0.99		
8	Solyc08g080140.4.1	A0A3Q7HT77	bifunctional dTDP-4-dehydrorhamnose 3,5-epimerase/reductase	−1.72	−1.65	-
58	Solyc09g009020.3.1	P26300	enolase		−1.15	
59	Solyc04g011510.4.1	A0A3Q7FZI5	triosephosphate isomerase		−1.28	
60	Solyc10g085550.3.1	A0A3Q7IN81 *	enolase		−2.06	
**Polysaccaride Metabolism (5)**						
61	Solyc03g123630.4.1	A0A3Q7GM93 *	pectinesterase/pectinesterase inhibitor U1 precursor	0.61		
12	Solyc01g104950.4.1	A0A3Q7EQN7	beta-D-xylosidase 2 precursor	−0.74	−0.62	-
13	Solyc01g107590.3.1	A0A3Q7ESC5	cinnamyl alcohol dehydrogenase	−1.37		-
62	Solyc08g079080.5.1	A0A3Q7HT63	acid beta-fructofuranosidase		−2.56	
63	Solyc01g111230.3.1	A0A3Q7EVU4	dirigent protein		−3.00	
**Protein Metabolism (35)**						
64	Solyc06g072220.1.1	A0A3Q7GYL5	Kunitz trypsin inhibitor	1.50		
65	Solyc01g080010.2.1	A0A3Q7EHP2	xyloglucan endoglucanase inhibitor	0.95		
66	Solyc12g088670.2.1	O49877	cysteine protease CYP1	0.74		
67	Solyc09g007640.4.1	A0A3Q7HWG9	serine carboxypeptidase-like 50	0.67		
68	Solyc02g068740.3.1	A0A3Q7F1L3	glycine cleavage system H family	−0.58		
69	Solyc01g099760.3.1	A0A3Q7F894	LeMA-1 putatve Mg-dependent ATPase 1	−0.74	−0.74	
70	Solyc02g083710.3.1	A0A3Q7F6N7	26S proteasome non-ATPase regulatory subunit 4	−0.80		
71	Solyc04g076190.1.1	A0A3Q7G5A8	aspartic proteinase nepenthesin-1	−1.20		
72	Solyc04g080960.4.1	A0A3Q7GB74	pre-pro-cysteine proteinase	−1.23		
73	Solyc01g100320.3.1	A0A3Q7ENV3	disulfide-isomerase-like protein	−0.57		
74	Solyc07g049450.3.1	A0A3Q7HDI6	protein disulfide isomerase family	−0.61		
75	Solyc08g079170.3.1	A0A3Q7HV43	heat shock protein STI	−0.61		
76	Solyc08g079260.3.1	A0A3Q7HV74	tetratricopeptide repeat-containing	−0.63		
77	Solyc01g106260.3.1	A0A3Q7FBU5	heat shock protein 70	−0.64		
78	Solyc07g042250.3.1	Q9M5A8	chaperonin 21 precursor	−0.65		
79	Solyc01g088610.4.1	A0A3Q7F3J4	10 kDa chaperonin 1	−0.95		
80	Solyc06g065520.3.1	A0A3Q7GYZ2	T-complex protein eta subunit		−0.89	
81	Solyc03g121330.3.1	A0A3Q7FVJ9	60S ribosomal protein L28, putative	−0.61		
82	Solyc01g096580.3.1	A0A3Q7F6F2	ribosomal protein S10p/S20e	−0.62		
83	Solyc09g010100.3.1	Q2MI68 *	30S ribosomal protein S11	−0.63		
84	Solyc09g005720.3.1	A0A3Q7HVY6	60S ribosomal protein L23A	−0.69		
85	Solyc12g044720.2.1	A0A3Q7JA54	60S ribosomal L28-like protein	−0.69		
86	Solyc03g096360.4.1	A0A3Q7FPS6	60S ribosomal protein L35a-2	−0.70		
87	Solyc06g073430.4.1	A0A3Q7ITW7 *	40S ribosomal protein S29	−0.72		
88	Solyc11g017070.2.1	A0A3Q7JK86	eukaryotic translation initiation factor 3 subunit I	−0.73		
89	Solyc06g008170.3.1	K4CUW3	50S ribosomal protein L14	−0.80		
90	Solyc03g112360.1.1	A0A3Q7FRG1	60S ribosomal protein L27A	−0.82		
91	Solyc10g086010.2.1	A0A3Q7IMU3	60S ribosomal L4	−0.97		
22	Solyc01g099900.4.1	A0A3Q7ENE9	60S ribosomal protein L18-2	−0.99		+
92	Solyc01g099890.2.1	A0A3Q7ENE9 *	PUA domain-containing protein	−1.27		
93	Solyc12g096300.2.1	A0A3Q7JEJ5	40S ribosomal protein S6	−1.55		
94	Solyc07g005560.3.1	Q9AXQ5	eukaryotic translation initiation factor 5A		−0.84	
95	Solyc03g083390.4.1	A0A3Q7FN48	protein BOBBER 1	−0.66		
96	Solyc08g074290.3.1	A0A3Q7HSI3	myosin heavy chain-like protein	−0.86		
97	Solyc02g087300.1.1	A0A3Q7FW84	transducin/WD40 repeat-like	−1.00		
**Aminoacid Metabolism (9)**						
98	Solyc01g112280.3.1	A0A3Q7EV39	N-acyl-L-amino-acid amidohydrolase	0.61		
99	Solyc05g053810.3.1	A0A3Q7GNF6	serine hydroxymethyltransferase	−0.76	−0.76	
100	Solyc02g082830.3.1	A0A3Q7F688	phosphoserine aminotransferase 2	−0.93		
101	Solyc04g074480.3.1	A0A3Q7H097	3-deoxy-D-arabinoheptulosonate 7-phosphate (DAHP) synthase 2	−1.17		
28	Solyc09g008280.2.1	P43282	S-adenosyl-L-methionine synthase	−1.29		-
102	Solyc12g098490.2.1	A0A3Q7JE99	serine hydroxymethyltransferase	−1.32		
103	Solyc10g083970.1.1	A0A3Q7IMD9	S-adenosylmethionine synthase	−1.60	−1.00	
104	Solyc12g099000.3.1	A0A3Q7JEH3	S-adenosylmethionine synthase		−0.94	
105	Solyc04g076790.3.1	A0A3Q7G863	serine hydroxymethyltransferase		−1.84	
**Signaling/Regulation (11)**						
106	Solyc09g091000.4.1	A0A3Q7I801	pathogenesis-related protein STH-2	1.90		
107	Solyc09g082780.3.1	A0A3Q7I7U0	stem-specific protein TSJT1	0.64		
108	Solyc09g090990.2.1	A0A3Q7I9H4 *	major allergen Mal d 1		0.66	
109	Solyc12g088720.2.1	A0A3Q7JBV0	polyadenylate-binding protein	−0.62		
110	Solyc12g014210.3.1	A0A3Q7J5F6	UBP1-associated protein 2C-like	−0.62		
111	Solyc03g096460.4.1	Q672Q3	wound/stress protein precursor	−1.10		
112	Solyc04g074040.3.1	A0A3Q7H017	serine/arginine-rich splicing factor	−1.12		
113	Solyc02g071180.3.1	A0A3Q7F2T0	RNA polymerase II degradation factor-like protein (DUF1296)	−1.21		
114	Solyc09g009030.4.1	A0A3Q7HYW7	histone deacetylase HDT1	−1.46		
115	Solyc01g109660.2.1	A0A3Q7IGH7 *	glycine-rich RNA-binding protein		−0.62	
116	Solyc12g095960.3.1	A0A3Q7JDH7	Insulin-like growth factor 2 mRNA-binding protein 2		−0.92	
***Miscellaneous (3)***						
117	Solyc03g115110.4.1	A0A3Q7FRU0	ATP synthase subunit gamma	1.13		
118	Solyc06g062380.3.1	A0A3Q7GUF9	acid phosphatase-like protein 1	0.72		
119	Solyc11g039980.3.1	A0A3Q7JLN4	ATP synthase subunit alpha		0.63	

* When the accession sequence gave no hit in the Uniprot database, the closest homolog found in *Solanum lycopersicum* was used.

**Table 4:** Identified and quantified proteins (7 in total) affected by both Fe and Mn deficiencies. Accession code is from the ITAG 4.0 database. Fold changes correspond to the base 2 logarithm of the fold change between the corresponding deficient sample to the control samples. SG—Shotgun. 2D—2-DE electrophoresis. Relative abundance increases are underlined for the sake of clarity.

#	Accession	UniProtKB	Description	−Fe SG	−Fe 2D	−Mn SG	−Mn 2D
22	Solyc01g099900.4.1	A0A3Q7ENE9	ribosomal protein L18		1.51	−0.99	
2	Solyc12g005790.2.1	A0A3Q7JR84	peroxidase 27	−0.82		0.93	
1	Solyc07g052510.4.1	A0A3Q7HDZ4	peroxidase (TPX1)	−0.76	−3.00		−3.64
13	Solyc01g107590.3.1	A0A3Q7ESC5	cinnamyl alcohol dehydrogenase	−1.54		−1.37	
8	Solyc08g080140.4.1	A0A3Q7HT77	bifunctional dTDP-4-dehydrorhamnose 3,5-epimerase/reductase	−0.81		−1.72	−1.65
12	Solyc01g104950.4.1	A0A3Q7EQN7	beta-D-xylosidase 2	−0.90	−1.14	−0.74	−0.62
28	Solyc09g008280.2.1	P43282	S-adenosylmethionine synthase		−3.00	−1.29	

## Data Availability

Raw data is available in the ProteomeXchange Consortium via the Pride partner repository with the data set identifier PXD008326.

## Supplementary Materials

The following are available online at https://www.mdpi.com/article/10.3390/ijms23073719/s1.
