# Supplementary figures and images for: Effects of Fe and Mn Deficiencies on the Root Protein Profiles of Tomato (Solanum lycopersicum) Using Two-Dimensional Electrophoresis and Label-Free Shotgun Analyses
Source: Int J Mol Sci. 2022 Mar 28;23(7):3719. doi: 10.3390/ijms23073719 (PMC8998858; doi:10.3390/ijms23073719)

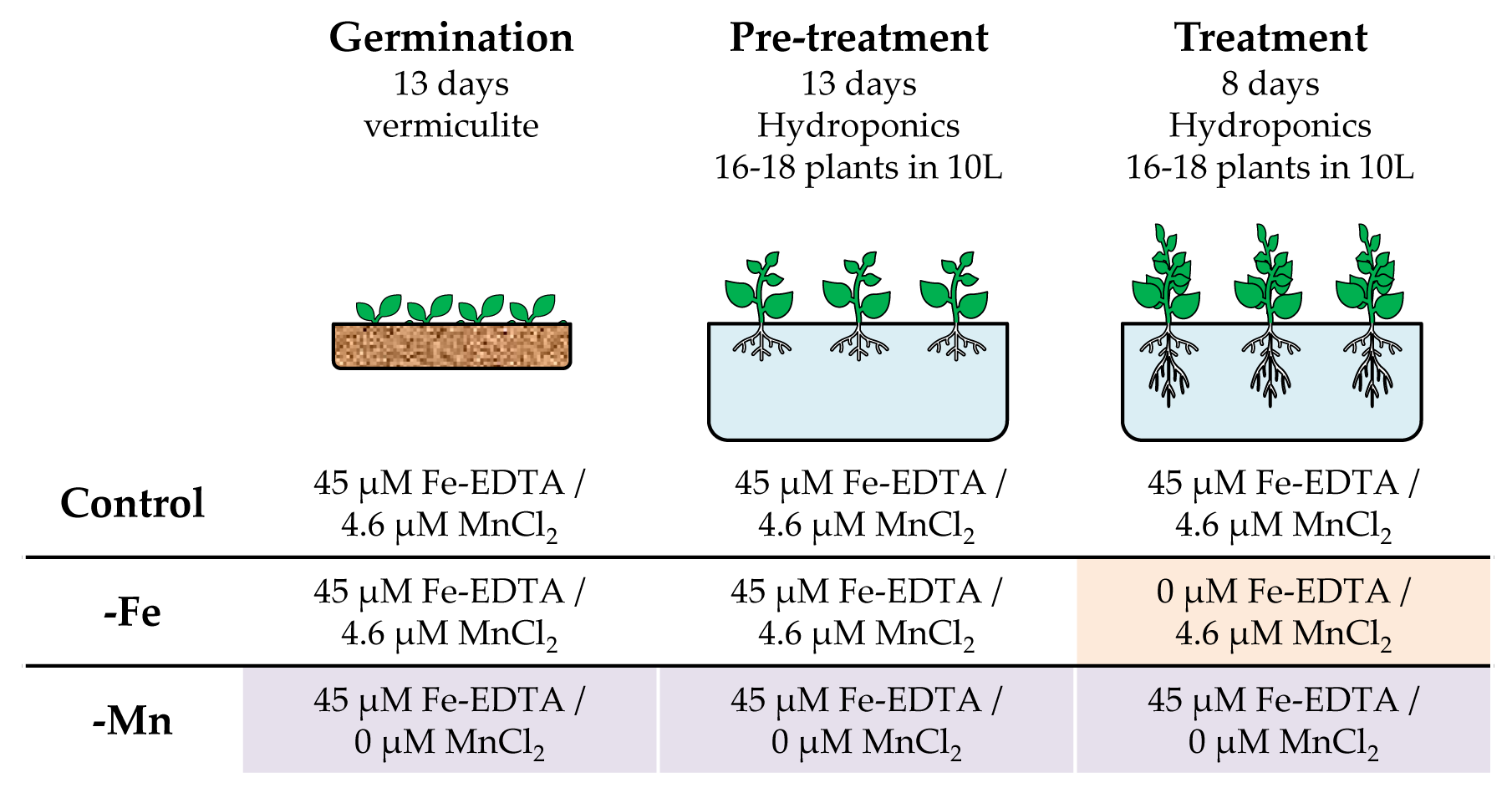

Supplement: Supplementary file 1 [file ijms-23-03719-s001.zip › FigureS1_New_R1.tif]
